# Supplementary material for: SARS-CoV-2 Selectively Induces the Expression of Unproductive Splicing Isoforms of Interferon, Class I MHC, and Splicing Machinery Genes
Source: Int J Mol Sci. 2024 May 23;25(11):5671. doi: 10.3390/ijms25115671 (PMC11172111; doi:10.3390/ijms25115671)
Supplement: Supplementary file 1 [file ijms-25-05671-s001.zip › Figure S1.pdf]

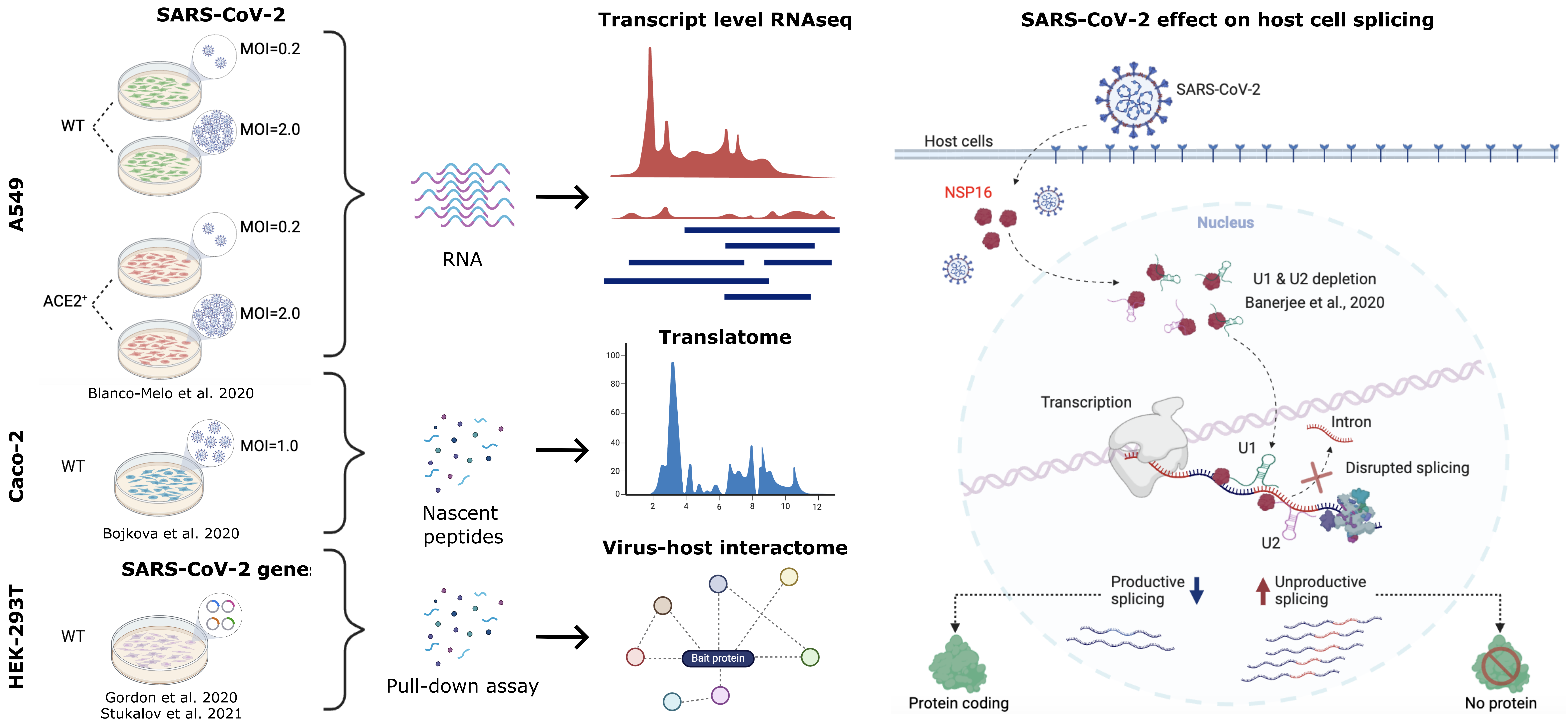

SARS-CoV-2
A549
WT
MOI=0.2
MOI=2.0
ACE2+
MOI=0.2
MOI=2.0
Blanco-Melo et al. 2020
Caco-2
WT
MOI=1.0
Bojkova et al. 2020
HEK-293T
SARS-CoV-2 gene
WT
Gordon et al. 2020
Stukalov et al. 2021
RNA
Nascent peptides
Pull-down assay
Transcript level RNAseq
Translatome
100
80
60
40
20
0
2
4
6
8
10
12
Virus-host interactome
Bait protein
SARS-CoV-2 effect on host cell splicing
SARS-CoV-2
Host cells
NSP16
Nucleus
U1 & U2 depletion
Banerjee et al., 2020
Transcription
Intron
U1
Disrupted splicing
U2
Productive splicing
Unproductive splicing
Protein coding
No protein
