# Supplementary material for: SARS-CoV-2 Selectively Induces the Expression of Unproductive Splicing Isoforms of Interferon, Class I MHC, and Splicing Machinery Genes
Source: Int J Mol Sci. 2024 May 23;25(11):5671. doi: 10.3390/ijms25115671 (PMC11172111; doi:10.3390/ijms25115671)
Supplement: Supplementary file 1 [file ijms-25-05671-s001.zip › ijms-2984655-supplementary.pdf]

Figure S1: Overview of all integrated datasets;
Figure S2: SARS-CoV-2 affects the expression of productive and unproductive transcripts in all cells;
Table S1: Differential Gene and Transcript expression results;
Table S2: fgsea results;
Table S3: Differentially Translated Transcripts;
Table S4: Gene set list used on functional enrichment.
