# Supplementary figures and images for: SARS-CoV-2 Selectively Induces the Expression of Unproductive Splicing Isoforms of Interferon, Class I MHC, and Splicing Machinery Genes
Source: Int J Mol Sci. 2024 May 23;25(11):5671. doi: 10.3390/ijms25115671 (PMC11172111; doi:10.3390/ijms25115671)

A

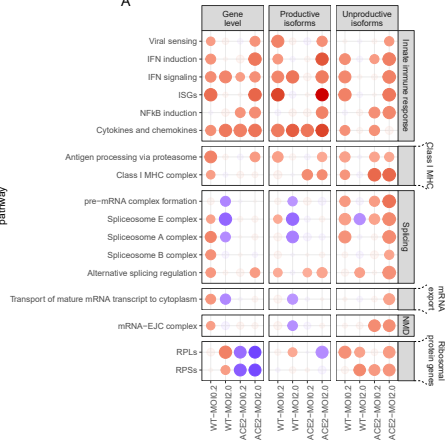

-log(pval)

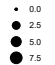

NES

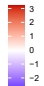

is.sig

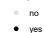

B

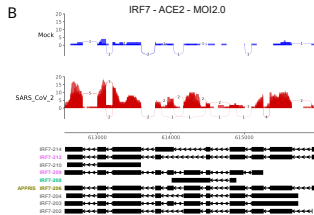

C

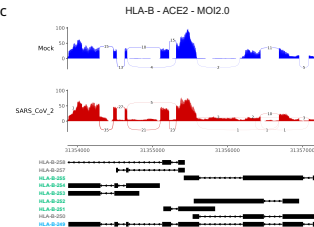

D

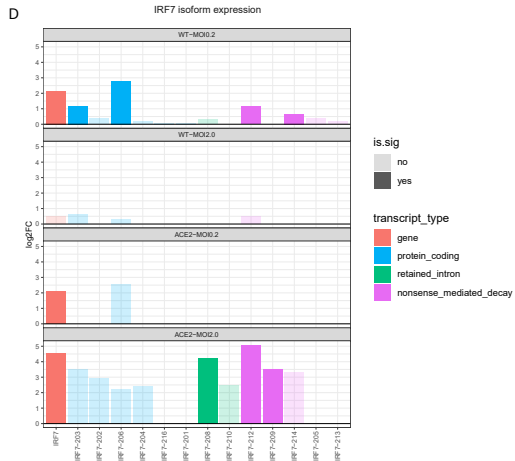

Supplement: Supplementary file 1 [file ijms-25-05671-s001.zip › Figure S2.pdf]
